# The Contribution to Stress Recovery and Attention Restoration Potential of Exposure to Urban Green Spaces in Low-Density Residential Areas

**DOI:** 10.3390/ijerph18168713

**Published:** 2021-08-18

**Authors:** Shuping Huang, Jinda Qi, Wei Li, Jianwen Dong, Cecil Konijnendijk van den Bosch

**Affiliations:** 1 College of Architecture and Planning, Fujian University of Technology, Fuzhou 350002, China; huangshuping@fjut.edu.cn; 2 School of Design and Environment, National University of Singapore, Singapore 117356, Singapore; 3 School of Art and Design, Zhengzhou University of Aeronautics, Zhengzhou 450015, China; lywvivin@126.com; 4 College of Landscape Architecture, Fujian Agriculture and Forestry University, Fuzhou 350002, China; fjdjw@fafu.edu.cn; 5 Faculty of Forestry, The University of British Columbia, Vancouver, BC V6T 1Z4, Canada; cecil.konijnendijk@ubc.ca

**Keywords:** mental health, psychological and physiological responses, landscape elements, perceived restorativeness scale, green spaces planning

## Abstract

This study assessed the contributions of urban green spaces on mental health with joint consideration of people’s physiological and psychological responses. The psychological and physiological responses of participants aged between 22 and 28, who visited green spaces in a low-density area of Fuzhou, Fujian Province, China, were measured using Perceived Restorativeness Scale (PRS) methods and biometric wearable sensing devices, respectively. Results showed that exposure to green space led to significant changes in PRS, electrodermal activity (EDA), facial electromyography (EMG), respiration sensor (RESP), and photoplethysmography (PPG), while there is no significant impact on skin temperature (SKT). Additionally, psychological and physiological responses were highly consistent and correlated (R < 0.8). The results also indicated that green spaces with high plant species richness, a water landscape, bumpy ground, cultural landscape, and without roadways presented a high performance on stress recovery and attention restoration. At the same time, the influence of openness was negligible in the low-density area. The study provides planners and landscape designers with specific guidance for implementing urban green spaces to improve mental health in low-density residential areas.

## 1. Introduction

The world’s urban population has rapidly increased from 0.8 to 4.2 billion between 1950 and 2018 and will continue to increase to an expected 5.7 billion in 2050 [1]. The rapid urbanization and associated urban sprawl destroy or modify natural areas (e.g., forestry and grasslands) in the process of transformation to buildings and human infrastructure to accommodate the growing urban population [2]. The decrease in natural areas in and near cities disconnects people from nature, something which has been found to contribute to stress-related health issues [3]. Green spaces (e.g., parks, gardens, and green streetscapes) have widely been employed to improve the urban environment and mental health, as access to urban nature and going outdoors are associated with positive psychological well-being [4].

Two theoretical perspectives are especially relevant to the positive psychological effect of green space on well-being [5]. The Attention Restoration Theory (ART) emphasizes the perceptive response of humans to the natural world, aiming to understand how people perceive, understand, and explore natural settings [6]. This theory inclines to stimulate people’s attention indirectly by characterizing feelings into four categories: being away, fascination, extent, and compatibility [7]. ART has been widely used to assess the psychological benefits of green spaces, particularly on relieving anxiety [8], reducing stress [9], increasing happiness [10], and restoring attention [3]. Comparatively, the psycho-evolutionary Stress Recovery Theory (SRT) can evaluate human restorative responses to natural environments directly, often within minutes [11]. This theory assumes that humans have two physiological responses to contact with nature: the preference for the natural environment and restoration after stressful events [12]. Based on this theory, several physiological indicators have been proposed to measure the human response of accessing the natural environment, including heart rates [13], blood pressure [8], muscle tension [14], and salivary cortisol level [13].

Just understanding the theories of stress recovery and attention restoration is not enough to support the improvement of the urban environment in practice due to the limited details provided to guide the provision, design, and management of green space. Therefore, studies have attempted to identify the specific elements and configurations of green space that have significant impacts on mental health. For example, planting trees in barren areas leads to a significant increase in stress recovery, while the performance varies across the vegetation density and species [15]. Hoyle et al. [16] found that native plants with multiple layers were perceived as significantly more restorative than non-native plants with a single layer. The performance of stress recovery may further be improved by viewing pleasant rural scenes [17]. Additionally, water bodies are a highly valuable element in stress recovery [18]. White et al. [19] pointed out that those with access to water bodies suffer lower levels of stress. However, the impacts of the water intervention (e.g., waterbody types and waterbody size) in green space on stress recovery are still unclear due to the limited amount of studies to date. Additionally, animals (e.g., birds) in green spaces help reduce levels of stress and anxiety since they can provide humans with a source of companionship, support, and entertainment [20,21]. Other elements, including openness [22], aesthetics [23], fascination [24], and accessibility [25,26], have also been discussed to understand the impacts of green space on stress recovery and attention restoration.

Based on existing findings, a set of projects have been proposed by governments or organizations to improve and green urban environments. For example, URBACT introduced a Health and Greenspace action to link the green infrastructure design and management to urban health policies and practices in order to promote mental and physical health for communities [27]. The World Health Organization (WHO) developed an urban green space indicator and toolkit to help policy-makers with evidence-based green space interventions for the health promotion of urban residents [28]. Although these greening projects present useful suggestions and guidance for developing healthy cities, the realization and design of green spaces can be challenging for compact urban areas in practice due to limited available space and intense competition with other land use [29]. Alternatively, greening the low-density urban areas can be an option for improving urban environments. In countries such as China, low-density areas have more easily available land for greening while still hosting a substantial number of people. This points to their feasibility for green space development in light of the need for mental health improvement. However, due to a lack of studies specifically focusing on green spaces in low-density urban areas and the different characteristics between compact and low-density areas, whether low-density residents’ exposure to green space can positively influence their mental health is still not clear. Therefore, there is a need to understand the impacts of green spaces on stress recovery and attention restoration in low-density urban areas.

Additionally, existing studies often assess stress recovery and attention restoration from a single perspective, which can be either physiological or psychological. On the one hand, psychological assessment based on surveys and investigation mainly emphasizes human perception. Grahn and Stigsdotter [30] employed perceived sensory dimensions (PSD) to assess the stress restoration of green space via investigating 953 participants in Sweden. Wang, Zhao, Meitner, Hu, and Xu [23] utilized the perceived restorative scale (PRS) to assess the restorative potential and aesthetic preference of green space. The Restoration Outcome Scale (ROS) is used by Mattila et al. [31] to explore the restorative experiences in a virtual reality forest environment. The results of these studies strongly depend on people’s voluntary participation and suffer from subjectivity and bias. On the other hand, physiological assessment enables detecting human responses to green space more objectively. Physiological indicators such as heart rate variability (HRV), skin temperature (SKT), and electroencephalography (EEG) have been used to measure the stress recovery of green façade [32]. Li et al. [33] employed the skin conductance level (SCL) and blood volume pulse (BVP) to measure the restorative potential of participants viewing a virtual reality forest. However, physiological assessment fails to capture people’s feelings. Due to the complementary function of physiological and psychological assessment, they should be considered in a more integrated way, combining them into an assessment of restorative perceptions.

To help fill the gaps in research mentioned above, this study integrates physiological and psychological assessments to understand the contributions of urban green spaces to stress recovery and attention restoration in low-density residential areas. Specifically, the objectives of this study were (1) to measure and compare the physiological and psychological responses to accessible green spaces, and (2) to identify the key elements of green spaces that have significant influences on stress recovery and attention restoration in low-density areas. This study aimed to broader our understanding of the impact of green spaces on mental health improvement, thus facilitating the planning and design of green spaces in low-density residential areas. The remainder of this paper is structured as follows. Section 2 introduces the study area, participant groups, assessment, and statistical methods with details. Section 3 explains the physiological or psychological assessment results. Section 4 discusses the implication and limitations of this study, followed by a conclusion in Section 5.

## 2. Methods

### 2.1. Study Sites

This study was conducted in Fuzhou, Fujian Province, China. Fuzhou is a coastal city with 7.8 million people in 2019. The city experienced rapid urbanization in the past decade. According to the Fuzhou Municipal Bureau of Statistics (FMBS), the urban population was increased from 2.97 million in 2011 to 5.5 million in 2019 [34]. The population expansion accompanies increased demand for green spaces that can provide numerous benefits such as leisure purposes and stress recovery. However, the growth of land for accommodation purposes in Fuzhou affects the available land for urban green spaces. As a result, the development areas of urban green spaces was significantly decreased from 710 ha in 2010 to 166.86 ha in 2019 [34]. Alternatively, intensive green spaces have been dispersedly developed outside the compact urban area in the urban fringe, collectively called ‘low-density residential areas’ (or small–medium-sized cities) [35].

Linpu has almost 7000 people and an area of 154.72 ha, which is a typical low-density residential suburb (45.24 people/ha) in the southeast of Fuzhou city. This area has longitude and latitude by 119°21′45″ E–119°22′16″ E and 26°01′07″ N–26°01′28″ N, respectively. Linpu was selected as a case study for three reasons. First, there are several green spaces and water bodies in the area, which can provide an opening view and a thermal comfort environment. The high-quality landscape provides a tremendous restorative opportunity for children, elders, and workers, which is highly appropriate for investigating the impacts of green spaces on stress recovery and attention restoration. Second, Linpu has a large number of well-preserved historical buildings and cultures. Due to its historical values, the area was nominated to the seventh batch of ‘Chinese historical and cultural villages’ in 2019. The well-preserved heritage can represent the traditional culture of Fuzhou city. Finally, Linpu is close to several industrial parks (e.g., Strait International Conference and Exhibition Center), which implies that there is a need to improve the mental health of office workers.

**Figure 1:**
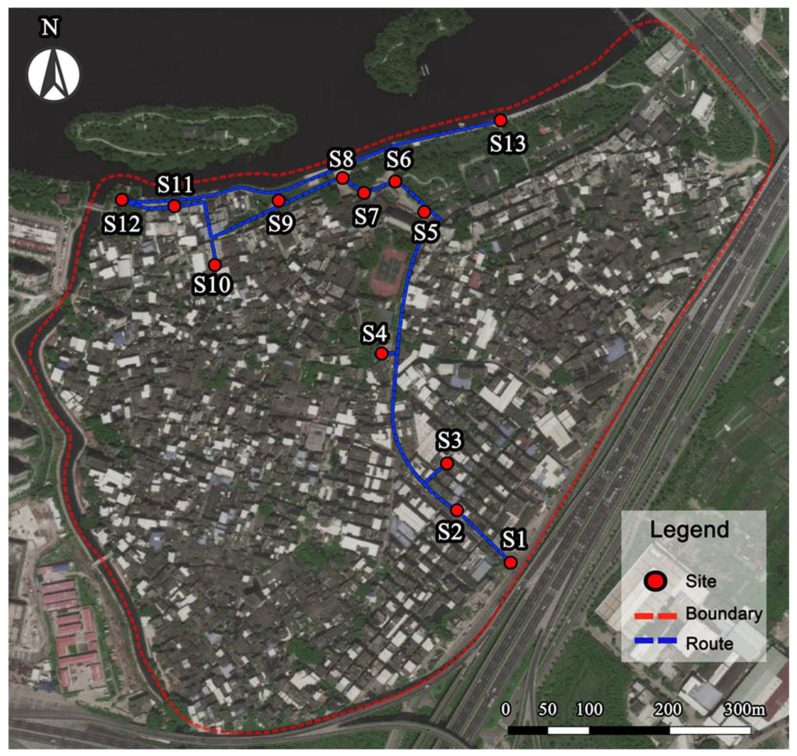
Aerial image of the Linpu experiment area redrawn based on Google map.

This study selected 13 typical sites of Linpu as experimental areas. The selected sites are further characterized based on plant richness, water landscape, openness, topography, road network, and cultural landscape to understand the impact of landscape elements on stress recovery. The details of these case areas and site attribution are illustrated in Figure 1 and Table 1.

### 2.2. Participants

The participants identified in the experiment were recruited by using two methods: advertisements through social media apps and face-to-face at the campus of Fujian Agriculture and Forestry University. The following inclusion criteria were used:

- People who have a mental illness history and undergo treatment for any disease are not included as they may feel depression and anxiety [32,37].
- Participants should have normal or corrected-to-normal vision since the loss of vision experiences continuous mental stress [32,38].
- People in the menstrual period are not selected since they may experience significant emotional changes caused by fluctuating hormone levels during this period [32,39].
- People with smoking or drinking habits are excluded because the habits can change feelings of depression, anxiety, and mood [37,40,41].

To avoid some interventions such as political stance, all selected participants were neither involved in the projects nor conflicted with interest. As a result, a total number of 33 participants consisting of 14 males and 19 females are identified for the experiment. The age of all participants ranged from 22 to 28 years old. All participants were students and visited all selected green spaces. The first perceptions of participants were employed to measure the effectiveness of each site on stress recovery and attention restoration.

### 2.3. Measurement of Physiological and Psychological Responses

Due to the different characteristics of physiological and psychological responses, the ways of measuring these are different. The following sections present the respective assessment methods used.

#### 2.3.1. Physiological Assessment

**Figure 2:**
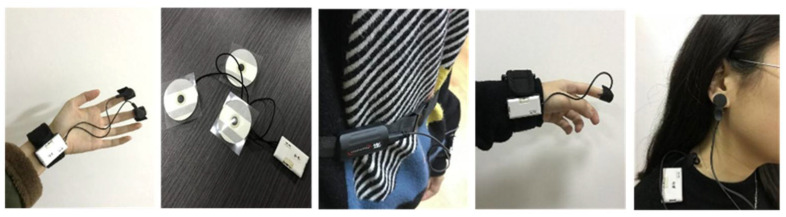
Biometric wearable sensing devices for measuring EDA, EMG, RESP, SKT, PPG.

Several indicators have been proposed to measure physiological responses, e.g., to green space use. In the present study, five widely used indicators, including electrodermal activity (EDA), facial electromyography (EMG), respiration sensor (RESP), skin temperature (SKT), and photoplethysmography (PPG), were selected to measure human emotional experiences. The values of indicators were measured by the ErgoLab data platform Version 2.0, which is a set of biometric wearable sensing devices, as shown in Figure 2 [42].

EDA aims to measure stress levels by monitoring skin conductance. Skin conductance caused by a stimulus of the sweat glands’ secretion was monitored by a wearable sensor with two reusable electrodes attached to two fingers of one hand [32]. High skin conductance leads to a high EDA, indicating the high stress levels of human bodies and negative physiological responses.

EMG reveals the emotional response by monitoring facial muscle activity. To ensure the accuracy and comprehensiveness of measurement, multiple sensors were employed to detect and amplify the small electrical impulses generated by specific facial muscles (e.g., corrugator and zygomatic muscles). The electrical impulses were then converted into EMG signals to measure facial muscle activity. A high value of the EMG signal means a strong reaction and high stress.

RESP aims to reveal the level of relaxation. A respiration sensor can measure the respiration waveform, amplitude, and frequency of the human body by detecting chest or abdominal expansion and contraction. The real-time information recorded by the sensor was inputted into a respiration belt place around the abdomen or chest for the calculation of RESP. A high RESP represents tension or nervousness, while low RESP represents relaxation [43].

SKT measures human stress by monitoring the skin temperature of fingers. Wireless skin temperature sensors attached to fingers were used to measure the SKT values. In general, low SKT can be monitored in a relaxed environment.

PPG reflects an emotional response by measuring heart rate variability. Heart rate variability caused by the surrounding environment was measured by a portable, wireless photoplethysmography sensor attached to the earlobe. High PPG commonly accompanies tension or nervousness.

#### 2.3.2. Psychological Assessment

Since PRS supports our understanding of stress reduction, the attraction of green space, the motivation for visiting, and the compatibility between people and nature, it has been widely used to measure the restorative quality of physical environments. This study employed a version of the PRS derived from Hartig, Korpela, Evans, and Gärling [7]. There are four scales and 26 items in the PRS, as shown in Table 2. Most items are evaluated with a 5-point scale from 1 = Not at all to 5 = Completely. However, some reverse items are assessed with a 5-point scale from 5 = Not at all to 1 = Completely due to their negative relation with restoration. The scores of all items are summed up to calculate the value of PRS. In general, high values indicate the high effectiveness of attention restoration.

### 2.4. Procedure

**Figure 3:**
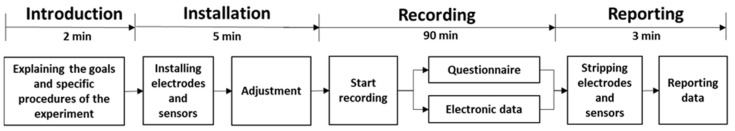
Process and timeline of assessments.

The experiments were conducted on sunny days from January 2018 to December 2018. The time of testing ranged from 8:00 to 11:00 and 14:00 to 17:00 in each experiment day. Figure 3 illustrates the process and time distribution of measurement. The measurement started with a brief introduction to explain the goals and specific procedures of the experiment. Then, the electrodes and sensors were installed on the participants. After a 2-min adjustment period, participants were asked to sit and rest for 3 min, followed by recording the physiological and psychological responses of participates in the village entrance as a baseline. Subsequently, participants visited the case areas from S1 to S13 in sequence. At each site, participants were given 5 min to fill a questionnaire (PRS) to test their attention level. Meanwhile, the electronic data were continually recorded during this period. Finally, the electrodes were stripped if data collection was finished, followed by reporting data.

### 2.5. Statistical Analysis

To ensure the reliability of electronic data, the Kruskal–Wallis (K–W) test was used to analyze the significance of EDA, EMG, RESP, SKT, and PPG. Meanwhile, the scores of PRS before and after the recovery stage were analyzed by Paired sample t-tests to keep the internal consistency. The level of significance was set at *p* < 0.05. The statistical analysis in this study was processed in IBM SPSS Statistics 22.

## 3. Results

### 3.1. Effects of Green Space on Stress Recovery

Results of the Kruskal–Wallis test show that EDA (*p* = 0.00), EMG (*p* = 0.00), RESP (*p* = 0.00), and PPG (*p* = 0.00) reached high levels of significance. Comparatively, SKT (*p* = 0.503 > 0.05) was not significantly different and was not be considered in stress reduction analysis.

Table 3 shows the average EDA, EMG, RESP, and PPG for the 13 sites. Sites with low values indicate the high performance of stress recovery. The results showed that the ranks of EDA, EMG, RESP, and PPG are the same. Specifically, S8 has the lowest value of EDA (2.62 μs), EMG (23.94 μV), RESP (8.48 rpm), and PPG (29.71 bpm), indicating the high potential on reducing perceived stress. In comparison, the numbers for S1 (EDA = 5.09 μs, EMG = 31.06 μV, RESP = 14.12 rpm and PPG = 30.10 bpm) and S3 (EDA = 4.78 μs, EMG = 30.35 μV, RESP = 13.63 rpm and PPG = 30.07 bpm) were found to be significantly larger than other sites, revealing the higher level of stress that participant experienced when visiting these sites.

### 3.2. Effects of Green Spaces on Attention Restoration

The results of paired sample t-tests show that all scales of PRS reached highly significant levels. Table 4 illustrates the average and rank of PRS. In general, the pattern of being away, fascination, extent, and compatibility are similar despite the inconsistency on some sites such as S10. A combination of these four indicators showed that the largest value was for S8 (3.77), followed by S7 (3.70). The value of S8 and S7 were considerably higher than those of other sites, which showed their high capacity for attention restoration, thanks to the high performance of S8 on stress reduction (Being Away = 3.59), motivation for visiting (Extent = 3.91), and a close relationship between human and nature (Compatibility = 3.63), as well as a great attraction of S7 (Fascination = 3.95). Comparatively, the lower value of S1 (Being Away = 1.64, Fascination = 1.96, Extent = 2.48, and Compatibility = 2.19) and S3 (Being Away = 1.95, Fascination = 2.57, Extent = 2.6, and Compatibility = 2.41) make them less suitable for attention restoration.

### 3.3. The Relationship between Stress Recovery and Attention Restoration

Pearson correlations were adopted to explore the bivariate relationships between stress reduction and attention restoration. Results showed that correlations between physiological and psychological were significant (*p* < 0.01). Table 5 depicts the specific correlations between these indicators. There is a strong correlation between physiological and psychological indicators, with R-values ranging from 0.827 to 0.977. Meanwhile, the interrelation of physiological and psychological indicators was also found to be strong (R > 0.822), especially for EMG and PPG (R = 0.999). Therefore, the impacts of exposure to urban green spaces on stress reduction and attention restoration are highly consistent.

### 3.4. Impact of Demographic Characteristics on Stress Recovery and Attention Restoration Potential

The participants are characterized according to their genders and ages in order to understand the impacts of demographic characteristics on the mental health improvement potentials of green spaces, as illustrated in Table 6. Results showed that males generally had lower physiological responses (e.g., EDA) and higher psychological reactions (e.g., Being Away) than females, indicating the higher performance on stress recovery and attention restoration potential achieved by males. Regarding the age, all participants are classified into two groups for analysis. Participants 22–25 years old generally have higher physiological responses than those 26–28 years old, despite the slightly lower indictors of RESP. However, the former has a lower psychological reaction than the latter, in particular with Being Away. The results indicated that visiting green spaces provided old groups (26–28) with less stress recovery and more attention restoration potential than young groups (22–25).

### 3.5. Impacts of Landscape Elements on Stress Recovery and Attention Restoration

**Figure 4:**
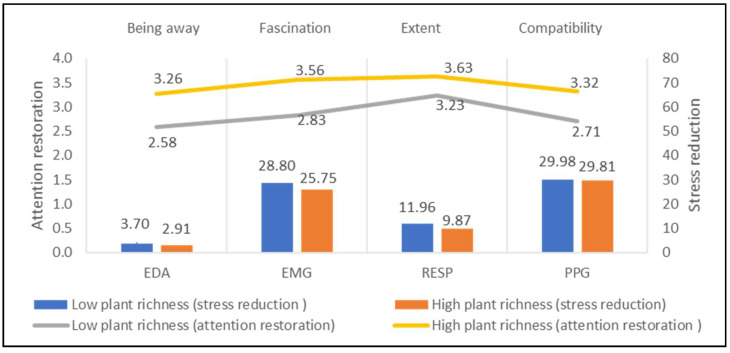
Impacts of plant richness on stress recovery and attention restoration.

This study characterized the 13 sites into different categories according to landscape elements, including plant richness, water landscape, openness, topography, road network, and cultural landscape. As almost all sites have vegetation, it is not appropriate to discuss the impact of vegetation and non-vegetation sites on mental stress. Alternatively, plant species richness is compared to understand the intervention of vegetation on mental stress. This study compared the physiological and psychological indicators of the seven study sites with low plant richness (plant species ≤ 3) and six study sites with high plant richness (plant species > 3). Figure 4 shows that sites with high plant richness have low physiological value and high psychological value than ones with low plant richness, particularly on EMG (difference = 3.05 μV) and fascination (difference = 0.73), indicating the higher potentials of rich species on stress reduction and attention restoration.

**Figure 5:**
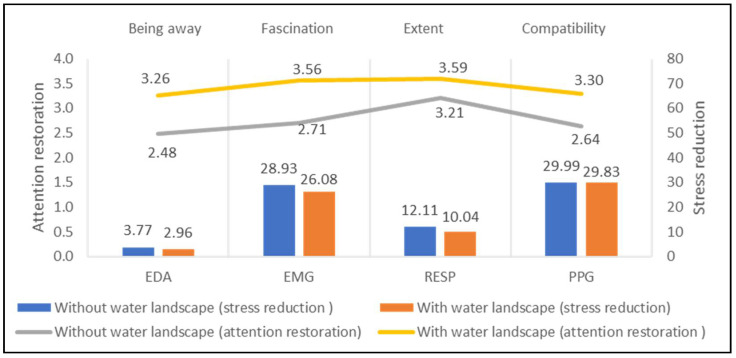
Impacts of water landscape on stress recovery and attention restoration.

Figure 5 shows the differences between the sites with water elements (seven study sites) and without water elements (six study sites) on physiological signals. The former has significantly lower values than the latter on EDA (difference = 0.81 μs) and EMG (difference = 2.85 μV), RESP (difference = 2.07 rpm), while the PPG of them is quite similar. Meanwhile, the attention restoration of sites with water (Being Away = 3.26, Fascination = 3.56, Extent = 3.63, Compatibility = 3.32) are higher than that ones without water (Being Away = 2.58, Fascination = 2.83, Extent = 3.23, Compatibility = 2.71). The results show a positive impact of water on stress reduction and attention restoration.

**Figure 6:**
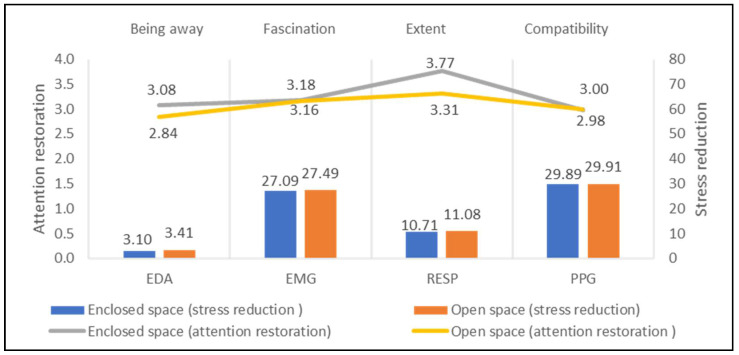
Impacts of space openness on stress recovery and attention restoration.

The physiological signals of enclosed spaces (3 study sites) and open spaces (10 study sites) are quite similar, as shown in Figure 6. Although the value of EDA, EMG, RESP, and PPG at enclosed space sites was found to be slightly lower than that at open spaces, their difference is quite narrow, with a value of no more than 0.4. Similarly, the difference of attention restoration between enclosed and open spaces is not significant, particularly on Fascination and Compatibility. Therefore, space openness possibly is not a vital landscape element for mental stress reduction and attention restoration in low-density contexts. 

**Figure 7:**
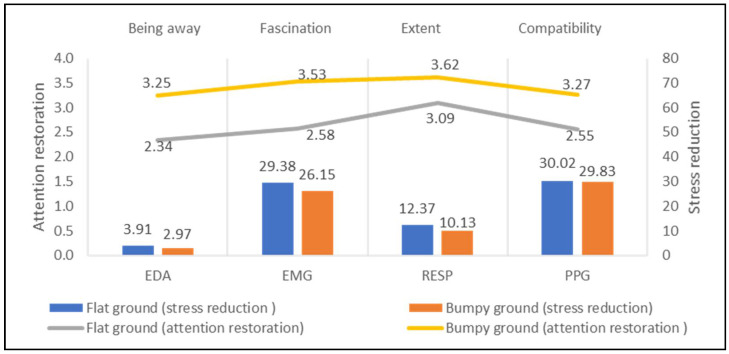
Impacts of topography on stress recovery and attention restoration.

Figure 7 shows that the sites with bumpy grounds (eight study sites) presented a higher performance on stress recovery and attention restoration than the ones with flat grounds (five study sites), particularly on EMG (difference = 3.23 μV) and RESP (difference = 2.24 rpm). Sites with bumpy grounds present higher values of attention restoration than ones with flat grounds, especially for fascination. Therefore, the topography is a key landscape element for stress reduction and attention restoration that should be considered in creating a well-being environment.

**Figure 8:**
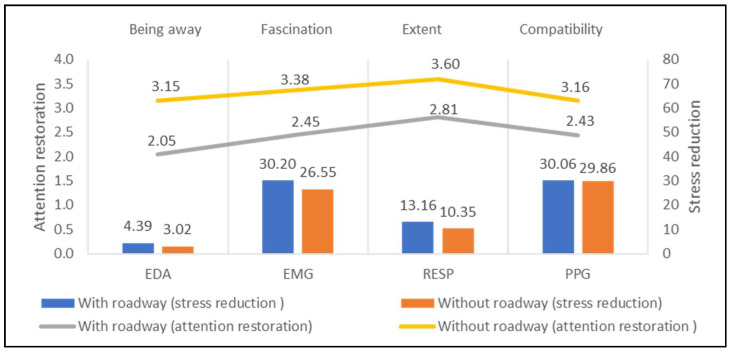
Impacts of the roadway on stress recovery and attention restoration.

Figure 8 compares the average performance of the 3 study sites with roadways (or driveway sites) and the 10 study sites without roadways (or non-driveway sites) related to attention restoration and stress reduction. Participants close to roadways experience high pressure on EDA (4.39 μs), EMG (30.20 μV) and RESP (13.16 rpm), while the figures are reduced to 3.02 μs, 26.55 μV, and 10.35 rpm, respectively, when they visited the sites without roadways. Comparatively, sites without roadways present higher psychological values (Being Away = 1.15, Fascination = 0.93, Extent = 0.79, Compatibility = 0.73) than roadways sites.

**Figure 9:**
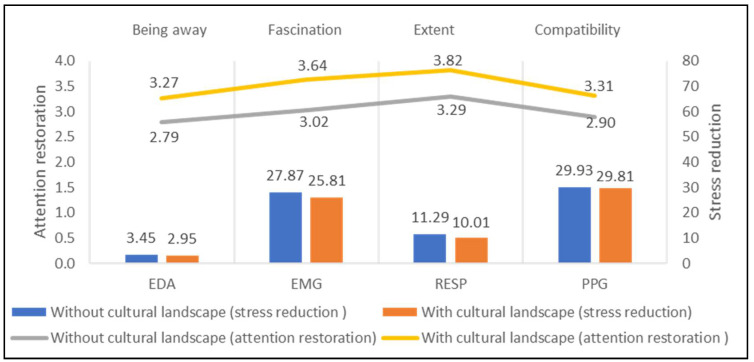
Impacts of cultural landscape on stress recovery and attention restoration.

Figure 9 depicts the average attention restoration and stress reduction of 3 study sites with cultural landscape and 10 study sites without cultural landscape. Sites with cultural landscapes (Being Away = 3.27, Fascination = 3.64, Extent = 3.82, Compatibility = 3.31) present greater attention restoration than those without cultural landscapes (Being Away = 2.79, Fascination = 3.02, Extent = 3.29, Compatibility = 2.90). Meanwhile, the former has lower physiological signals than the latter. The difference between cultural and non-cultural sites can reach 2.06 μV on EMG and 0.62 on fascination. Therefore, the display of cultural landscapes in green spaces can lead to low stress levels and high attention restoration.

## 4. Discussion

### 4.1. Physiological and Psychological Indicators to Measure Nature’s Restorative Effects

As mentioned, restorative experiences can be explained by two important theoretical frameworks: attention restoration theory and stress recovery theory [44]. Attention restoration can be generally evaluated by Being Away, Fascination, Extent, and Compatibility, which have been widely applied in restorative effects of green spaces from aesthetic appreciation [23,45] and acoustic characteristics [46]. Stress recovery can be measured by several existing methods, including individuals’ levels of stress [26] and the perceived restorative scale [23]. Studies on both attention restoration and stress recovery are based on questionnaires of which the accuracy highly relies on the personal preference, sample size, and selection of participants. If these psychological evaluations are integrated with physiological measurement to understand the restorative effects of green spaces, the accuracy can significantly be improved.

Physiological indicators have recently been adopted to monitor mental stress. For example, Cho et al. [47] measured EDA, PPG, and SKT to understand the information about stress levels and demonstrated the accuracy and reliability of physiological signals on stress measurement. The results showed that EDA and PPG are capable of reflecting the stress change, while SKT did not have a significant correlation with mental stress. This is consistent with our study. Larsen et al. [48] pointed out that pleasant stimuli present higher EMG activity over zygomaticus and less activity over corrugator supercilia, indicating the potential to evaluate the attraction of green spaces. The applicability of these indicators for understanding the effects of green space on mental stress has been fully demonstrated in this study.

However, these indicators are not enough to understand the impacts of green space on human well-being, and more physiological indicators should be adapted to explore the respective intervention. For example, heart rate variability related to sympathetic/parasympathetic nerves could be a potential indicator to understand the compatibility between visitors and green spaces [49]. Electrocardiographs aiming to reflect vagal and sympathetic nervous activity can support the upstanding of relaxed, exciting, and tensional conditions [50]. Electromyography has the potential to assess outdoor thermal comfort [51].

### 4.2. Stress Recovery and Attention Restoration of Different Gender and Age Groups

This study concluded that stress recovery and attention restoration of green spaces varied across the participants’ characteristics. The results indicated slightly higher restorative potentials of green spaces for males than for females. Similar conclusions have been found in Jiang et al. [52], who explore the relationships between tree cover, stress reduction, and gender differences. The results showed that increasing tree cover density could reduce male stress, but there is no relationship between females’ stress reduction and tree density. A potential explanation is the higher level of cortisol response to stress for males than females [52]. However, the gender difference is negligible in some cases. For example, Jiang et al. [53] also point to no correlation between genders and the impact of viewing green street scenes on stress recovery. This is possibly attributed to the difference in participants’ characteristics since the interactions of genders and demographic characteristics such as age can influence stress recovery potential [54].

The physiological and psychological responses of different age groups are different in this study. The attention restoration potential of young groups (22–25) is higher than the old group (26–28). This aligns with the finding that participants near green spaces with the age range of 18–24 have better mental health than those with the age range of 25–34 [54]. However, the old group (26–28) achieved a higher stress recovery potential than the young groups (22–25). The inconsistency between physiological and psychological responses can be explained by their different assessment approaches since the physiological responses and self-report results are different [54].

### 4.3. Characterizations of Green Spaces Based on Stress Recovery and Attention Restoration

This study concluded that plant richness, water landscape, topography, road network, and cultural landscape could significantly affect the performance of green spaces, while the influence of space openness was negligible. Specifically, this study found that using high plant richness presented higher potentials for stress reduction and attention restoration than low plant richness. The result is consistent with Navarrete-Hernandez and Laffan [55] that the diversity of vegetation was linked to mental stress reduction. It is also demonstrated by Huang et al. [56], who pointed out that the environment with grass had a significant positive impact on restoration. These results are possibly attributed to the fact that abundant vegetation invokes powerful emotional and spiritual experiences [57], increases visual complexity [58,59], and improves the aesthetic quality [60], thereby creating a favorite place and comfortable environment for visitors.

Green spaces with water bodies are more restorative than those without water bodies. The result aligns with Wang, Zhao, Meitner, Hu, and Xu [23], who found that access to clean water could improve both aesthetic preference and restoration. Similarly, White, Alcock, Wheeler, and Depledge [19] pointed out that people who lived in coastal areas suffered less stress than those in inland areas. The positive impacts of water can be explained by that water could satisfy the biological needs of human bodies and evoke a positive psychological response [61].

Green spaces with bumpy or uneven grounds defined as high surface variation within a certain elevation range are beneficial for stress reduction and attention restoration [36]. Similar results provided by Deng et al. [62] revealed that topography with a natural mountain forest presented high effectiveness on restorative effect. This is because topography can not only increase the stratification of landscapes and improve visual quality, thereby making participants happy and relax [23], but also change the height of sites, affecting noise propagation and, in turn, conducting a quiet environment [63].

This study also found that high stress and low attention recovery could be detected in the green spaces close to roadways. This result is inconsistent with Deng, Li, Luo, Fu, Ma, Sun, Huang, Cai, and Jia [62], who concluded that roads did not have significant impacts on psychological restoration. The inconsistency can be explained by the different locations of roads. In the study of Deng, Li, Luo, Fu, Ma, Sun, Huang, Cai, and Jia [62], the majority of roads located in the green spaces were walkways. Comparatively, roadways in this study near the boundary areas have heavy traffic. In other words, the sites closed to roadways may accompany a high volume of noise and vehicle exhaust, which increases the stress and negatively affects restoring attention [63].

Cultural landscapes in green spaces are beneficial for stress recovery. This is consistent with the findings of Boucher et al. [64] and Packer and Bond [65] that access to the historical heritage and the museum could provide restorative experiences. A possible explanation is that cultural landscapes provide people, especially local visitors, with perceptions of the learning experience and the emotional resonance to create ‘favorite places’, including both natural settings and social experience [66]. The places allow people to relax, calm down, or clear their minds, supporting relaxation and attention restoration [65]. The explanation can also be demonstrated by Gesler [67], that religious heritage presented strong symbolic meaning, which can be particularly therapeutic for community members.

Additionally, the influence of space openness on stress recovery and attention restoration was negligible in this study. This can be supported by Shi et al. [68], who concluded that moderate spaces with the appropriate enclosure are ambiguous and increase the arousal level since the spaces are more beneficial for creating understandable, clear, and comfortable environments. This means the effectiveness of open and enclosed spaces on stress recovery and attention restoration can be similar and lower than moderate spaces. However, due to the limited studies on the impacts of space openness on stress reduction and attention restoration, their specific relationship should be further explored.

### 4.4. Policy Implication

In the urban sprawl, low-density areas have more available lands to develop green spaces than compact urban areas, providing opportunities to apply our findings. To support the decision-making of green space in low-density areas, this study provides some suggestions for mental health improvement. First, more vegetation species are encouraged in the green spaces. The vegetation can be combinations of trees, hedges, and grass since the rich plant species richness can positively impact the stress recovery and attention restoration potential, aesthetic preferences and quality [69], and thermal improvement [70]. Second, green spaces planning should highlight water and cultural landscapes due to their positive impacts on mental health. It is recommended to provide sites for people to view the water and cultural landscapes and explore the uniqueness of culture to raise public awareness about water protection and culture promotion. Thrid, slightly modifying the topography of green spaces can improve people’s mental health since bumpy grounds are more beneficial for stress recovery and attention restoration than flat grounds. Finally, the roadway in green spaces should be reduced due to their negatives impacts on stress recovery and environmental problems such as pollutions and noise.

### 4.5. Limitations

Although this study was capable of measuring the physiological and psychological activities of green spaces in urban areas, it also had limitations. First, due to the high demand for participants and a great volume of time for training, the sample size was relatively small and possibly is not enough to represent the wider population. For example, the age group of participants 22 to 28 years old is narrow. Additionally, most selected participants are students. The results represent the mental health benefits of education-related groups, which may have potential bias. More participants covering an extensive group should be considered in future research. Second, this study only focuses on the first perceptions of people regarding the stress recovery of green spaces. However, the recovery ability, particularly with physiological responses, varies across repeated times and periods [71]. The contributions to mental health benefits of repeated exposure to green space should further be explored. Third, the effects of green spaces on physiological and psychological activities involve not only landscape elements but also other factors, e.g., aesthetic appreciation [23,72], soundscape characteristics of animals [46,73,74], and outdoor thermal environment [75,76]. Further consideration of these factors is required to understand the impacts of green spaces fully.

## 5. Conclusions

Understanding the impacts of green spaces on mental health improvement in urban areas, particularly in low-density residential areas, helps develop healthy cities. This study investigated the physiological and psychological responses of participants aged between 22 and 28 visiting green spaces in low-density areas. The results showed the significant impacts (*p* < 0.05) of green spaces on participants’ PRS, EDA, EMG, RESP, and PPG, despite the negligible influence on SKT. The psychological and physiological responses of participants were highly consistent and correlated (R < 0.8). Additionally, the typology of green spaces is discussed to provide suggestions for planning green spaces in low-density areas. Based on the results, this study recommended increasing plant species richness, water landscape, bumpy ground, and culture landscape, and reducing roadways to improve the stress recovery and attention restoration potential of green spaces. However, no significant correlation was found between the mental health benefits and openness of green spaces in low-density areas. The findings can demonstrate the positive impacts of green spaces on stress recovery and attention restoration and help design high-performance green spaces. Overall, this study provides planners and landscape designers with specific information on specific configurations and characteristics of green spaces in low-density areas that promote mental health, supporting their decision-making.

## Figures and Tables

**Table 1:** Characteristic of experiment sites in Linpu villageSite.

	Name	Image	Landscape Elements	Name	Types	Image	Landscape Elements
S1	Entrance	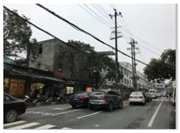	Low plant richness Without water landscape Open space Flat ground With roadway Without cultural landscape	S8	Riverside Plaza	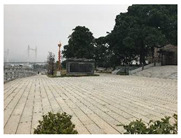	High plant richness With water landscape Open space Bumpy ground Without roadway Without cultural landscape
S2	Pocket green space	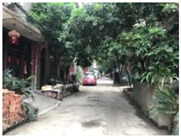	Low plant richness Without water landscape Open space Flat ground With roadway Without cultural landscape	S9	Promenade along the river	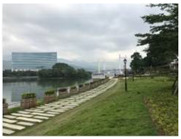	High plant richness With water landscape Open space Bumpy ground Without roadway Without cultural landscape
S3	Main road	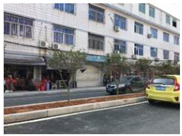	Low plant richness Without water landscape Open space Flat ground With roadway Without cultural landscape	S10	Pocket space	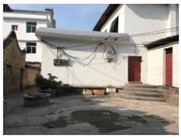	Low plant richness Without water landscape Enclosed space Flat ground Without roadway Without cultural landscape
S4	High Platform	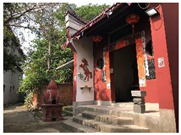	Low plant richness Without water landscape Enclosed space Bumpy ground Without roadway With cultural landscape	S11	Leisure Square	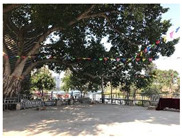	High plant richness With water landscape Open space Bumpy ground Without roadway Without cultural landscape
S5	School	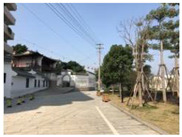	High plant richness Without water landscape Open space Flat ground Without roadway Without cultural landscape	S12	Fitness area	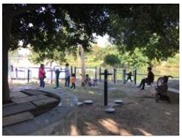	Low plant richness With water landscape Open space Bumpy ground Without roadway Without cultural landscape
S6	Academy	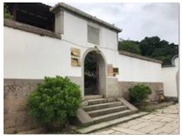	Low plant richness With water landscape Enclosed space Bumpy ground Without roadway With cultural landscape	S13	Waterfront Landscape Road	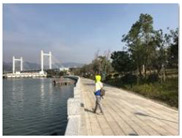	igh plant richness With water landscape Open space Bumpy ground Without roadway Without cultural landscape
S7	Banyan Square	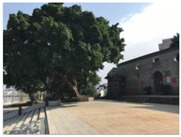	High plant richness Without water landscape Open space Bumpy ground Without roadway With cultural landscape				

Note: low and high plant richness refers to the number of plant species less and more than three, respectively [30]. Water landscape is the river, pool, fountain, or other water on the site. Open and enclosed spaces are the experience of open and closed surroundings for expression and activity [30]. Flat and bumpy ground refers to the low and high surface variation, respectively, within a certain elevation range [36]. The roadway in this study is defined as a road intended for vehicles. Culture landscape is the historical features and buildings on the site [30].

**Table 2:** The scales and items of perceived restorativeness scale methods.

No.	Items	Scale
1	Being here is an escape experience.	Being Away
2	Spending time here gives me a break from my day-to-day routine.	
3	It is a place to get away from it all.	
4	Being here helps me to relax my focus on getting things done.	
5	Coming here helps me to obtain relief from unwanted demands on my attention.	
6	This place has fascinating qualities.	Fascination
7	My attention is drawn to many interesting things.	
8	I want to get to know this place better.	
9	There is much to explore and discover here.	
10	I want to spend more time looking at the surroundings.	
11	This place is boring. *	
12	The setting is fascinating.	
13	There is nothing worth looking at here. *	
14	There is too much going on. *	Extent
15	It is a confusing place. *	
16	There is a great deal of distraction. *	
17	It is chaotic here. *	
18	Being here suits my personality.	Compatibility
19	I can do things I like here.	
20	I have a sense that I belong here.	
21	I can find ways to enjoy myself here.	
22	I have a sense of oneness with this setting.	
23	There are landmarks to help me move around.	
24	I could easily form a mental map of this place.	
25	It is easy to find my way around here.	
26	It is easy to see how things are organized.	

Note: * reverse items.

**Table 3:** Results of EDA, EMG, RESP, and PPG.

Site	EDA (μs)	Rank	EMG (μV)	Rank	RESP (rpm)	Rank	PPG (bpm)	Rank
S1	5.09	13	31.06	13	14.12	13	30.10	13
S2	3.31	10	29.18	10	11.73	10	30.01	10
S3	4.78	12	30.35	12	13.63	12	30.07	12
S4	3.07	6	26.66	6	10.77	6	29.87	6
S5	3.17	8	27.96	8	11.14	8	29.94	8
S6	3.02	5	26.24	5	10.11	5	29.83	5
S7	2.76	2	24.54	2	9.14	2	29.74	2
S8	2.62	1	23.94	1	8.84	1	29.71	1
S9	2.94	4	25.58	4	9.83	4	29.80	4
S10	3.22	9	28.37	9	11.24	9	29.97	9
S11	3.12	7	27.49	7	10.84	7	29.90	7
S12	3.42	11	29.74	11	12.14	11	30.04	11
S13	2.84	3	25.01	3	9.40	3	29.77	3

**Table 4:** Results of the assessment of psychological restoration using the Perceived Restorativeness Scale.

Site	Being Away	Rank	Fascination	Rank	Extent	Rank	Compatibility	Rank	Overall	Rank
S1	1.64	13	1.96	13	2.48	13	2.19	13	2.07	13
S2	2.57	11	2.83	10	3.34	9	2.7	10	2.86	10
S3	1.95	12	2.57	12	2.6	12	2.41	12	2.38	12
S4	3.19	5	3.36	6	3.8	4	3.04	6	3.35	6
S5	2.62	10	2.95	8	3.37	8	2.91	7	2.96	8
S6	3.16	6	3.6	5	3.83	3	3.34	5	3.48	5
S7	3.45	3	3.95	1	3.83	2	3.56	2	3.70	2
S8	3.59	1	3.94	2	3.91	1	3.63	1	3.77	1
S9	3.43	4	3.62	4	3.78	5	3.48	4	3.58	4
S10	2.9	8	2.58	11	3.67	7	2.56	11	2.93	9
S11	2.95	7	3.17	7	3.14	10	2.84	8	3.03	7
S12	2.67	9	2.89	9	2.89	11	2.72	9	2.79	11
S13	3.54	2	3.73	3	3.75	6	3.51	3	3.63	3

**Table 5:** Pearson correlations between stress recovery and attention restoration.

Indicators	EDA	EMG	RESP	PPG	Being Away	Fascination	Extent	Compatibility
EDA	1							
EMG	0.838 **	1						
RESP	0.935 **	0.974 **	1					
PPG	0.822 **	0.999 **	0.968 **	1				
Being Away	−0.938 **	−0.945 **	−0.977 **	−0.935 **	1			
Fascination	−0.843 **	−0.956 **	−0.944 **	−0.956 **	0.932 **	1		
Extent	−0.893 **	−0.889 **	−0.922 **	−0.873 **	0.920 **	0.827 **	1	
Compatibility	−0.827 **	−0.969 **	−0.952 **	−0.970 **	0.926 **	0.980 **	0.845 **	1

Note: ** *p* < 0.01.

**Table 6:** Stress recovery and attention restoration potential of different demographic characteristics.

Demographic Characteristics		EDA (μs)	EMG (μV)	RESP (rpm)	PPG (bpm)	Being Away	Fascination	Extent	Compatibility
**Gender**	Female	3.40	28.67	10.77	29.91	2.86	3.16	3.45	2.93
	Male	3.26	26.22	11.31	29.89	3.17	3.30	3.24	3.25
**Age**	22–25	3.39	27.91	10.85	29.93	2.86	3.23	3.40	2.99
	26–28	3.48	30.33	10.75	29.93	3.12	3.30	3.42	3.11
